# Indirect somatic embryogenesis of *Piper hispidinervum* L. and evaluation of the regenerated plants by flow cytometry

**DOI:** 10.1186/s43141-022-00323-6

**Published:** 2022-03-01

**Authors:** Paulo Cesar Alves de Sousa, Stênio Steferson Silva e Souza, Gabriela Ferreira Nogueira, Inaê Mariê de Araújo Silva-Cardoso, Jonny Everson Scherwinski-Pereira

**Affiliations:** 1 Universidade de Brasília, Campus Universitário Darcy Ribeiro, Brasília, DF 70910-900 Brazil; 2 Posdoctoral Fellows from the Conselho Nacional de Desenvolvimento Científico e Tecnológico (CNPq, Brasília, Brazil)/Embrapa Recursos Genéticos e Biotecnologia, Brasília, DF Brazil; 3 Embrapa Recursos Genéticos e Biotecnologia, Av. W5 Norte (final), Brasília, DF 70770-917 Brazil

**Keywords:** Piperaceae, Morphogenesis, Somatic embryos, Plant regeneration, Cellular DNA content

## Abstract

**Background:**

*Piper hispidinervum* is a species native from the Amazon region with great economic potential, given its scientifically proven insecticidal properties. In this study, an efficient protocol of plant regeneration via indirect somatic embryogenesis has been established for the first time. In a first experiment, for the induction of calluses, foliar explants of non-discriminated accesses of *P. hispidinervum* were inoculated in MS medium supplemented with α-naphtalenacetic acid (NAA) and 6-benzylaminopurine (BAP), in different combinations. For a second experiment, foliar explants from five different accesses of *P. hispidinervum* (PH17, PH21, PH28, PH37, and PH39) were analyzed regarding the formation of calluses when cultivated in MS medium with 5 mg L^−1^ NAA + 2.5 mg L^−1^ BAP. To obtain somatic embryos-like structures, calluses were cultivated in MS medium with 10 mg L^−1^ NAA + 2.5 mg L^−1^ of BAP. The somatic embryos-like structures obtained were inoculated in MS medium devoid of growth regulators and the plantlets were subjected to acclimatization. Calluses and somatic embryos-like structures were subjected to anatomical analysis and genetic stability of regenerated plants was analyzed by flow cytometry.

**Results:**

The treatments 2.5 mg L^−1^ BAP and 5 mg L^−1^ NAA + 2.5 mg L^−1^ BAP, after 60 days of cultivation, provided each 32% of primary callus, not being verified the formation of calluses in medium devoid of BAP. It was found that accesses differed among them with respect to the formation of primary calluses, with emphasis on accesses PH28, PH37, and PH39, with mean percentage of 95.3%. Regarding the percentage of embryogenic calluses and formation of somatic embryos-like structures, there were no statistical differences between accesses, with mean values of 90.6% and 77.3%, respectively. The somatic embryos-like structures of *P. hispidinervum* have conspicuous morphoanatomical similarities with the zygotic embryo, and flow cytometry analysis showed no significant variation in nuclear DNA size among plants regenerated in vitro and plants coming from seed germination, which indicates ploidy level stability.

**Conclusion:**

This protocol is the first cited in the literature that demonstrates an efficient micropropagation process by somatic embryogenesis of *P. hispidinervum.* It can be used either to enable large-scale vegetative production or to subsidize germplasm conservation or genetic engineering of *P. hispidinervum*.

## Background

The species of *Piper* are not only plants commonly used as seasonings in food, but are also producers of secondary metabolites with various biological effects on human health, as well as scientifically proven antibacterial, antifungal, and insecticidal properties [1].

Among these species, *Piper hispidinervum* C. DC., a species native to the Brazilian Amazon and popularly known as long pepper, has attracted the attention of the scientific community and producers due to its high safrole content [2], which can reach up to 97% [3]. Safrole, initially exploited in *Sassafras albidum* plants, nowadays threatened with extinction, is considered one of the most used essential oils in the world [4] and is a chemical used to synthesize piperonyl butoxide (PBO), a synergistic agent in the production of bioinsecticides [5]. In this scenario, *P. hispidinervum* has been attracting attention as an alternative and natural source of safrole [6].

It is important to emphasize that the insecticidal activity of *P. hispidinervum* has already been reported in *Sitophilus zeamais* [7], *Tenebrio molitor* [8], *Spodoptera frugiperda* [9], *Aedes aegypti*, *Tetranychus urticae*, and *Cerataphis lataniae* [10], as well as reports of antifungal activities [11], amoebicide [12], nematicide [13], and antimicrobial [14].

Despite the great potential for economic/industrial exploitation, *P. hispidinervum* is still in the process of domestication [15]. It should also be noted that long pepper is an allogamous species [16], with high genetic variability [17]. According to the latter authors, this species has great potential for obtaining varieties with high commercial value; however, it has a deficit of detailed studies regarding germplasm selection and propagation. In this context, Silva et al. [18] warn of the urgent need for research to establish a large-scale production system that enables its commercial planting, germplasm conservation studies and genetic manipulations.

Micropropagation arises, within this context, with a method that allows the clonal propagation of genotypes with characteristics of interest, such as high levels of safrole, and in a relatively short period. However, there are few reports of in vitro cultivation of *P. hispidinervum* aiming at propagation, being restricted, as far as our knowledge goes, to Silva et al. [18], who propagated long pepper plants by proliferation of lateral buds.

Among micropropagation techniques, somatic embryogenesis stands out as a crucial tool for biotechnological purposes, enabling in addition to clonal propagation, cryopreservation of germplasm and genetic transformation, including gene editing. It is worth emphasizing that *P. hipidinervum* is included in the list of priority species for conservation [19], which justifies the development of protocols that allow its conservation [20].

Despite all the benefits of somatic embryogenesis, plants regenerated from somatic embryos can exhibit somaclonal variation [21, 22]. Therefore, it is necessary to evaluate the stability of the genome of plants regenerated in vitro, especially when the goal is conservation of genetic resources and propagation of genotypes with specific characteristics obtained through breeding programs, which consume money and time [22]. Among the methods used to evaluate genome changes in plants cultivated in vitro, flow cytometry, considered a practical, reliable and fast technique for DNA ploidy screening, is cited [21], as well as reproducible and sensitive [23].

Within this context, the objective was to develop an efficient somatic embryogenesis protocol for *Piper hispidinervum* from foliar explants, in addition to evaluating the embryogenic potential of different accesses and genetic stability of regenerated plants.

## Methods

### Plant material

The plant material used consisted of foliar explants obtained from plants germinated in vitro of different *Piper hispidinervum* accesses (PH17, PH21, PH28, PH37, and PH39) selected for oil yield and safrole contents, from the Embrapa Acre Germplasm Bank, Rio Branco, Acre, Brazil.

Initially, the seeds of the different accesses were subjected to disinfestation, characterized by immersion for three minutes in 70% ethanol (v/v), followed by immersion for 20 min in sodium hypochlorite (NaOCl) (2.0–2.5% active chlorine) and four washings in distilled and autoclaved water. Then, the seeds were inoculated in vials containing 30 ml of MS culture medium [24] plus 30 g/l sucrose (Sigma, St. Louis, MO, USA). The pH of the medium was adjusted to 5.8 ± 0.1 prior to the addition of the gelling agent (2.5 g/l Phytagel–Sigma, St. Louis, MO, USA). Sterilization of the medium was carried out by autoclaving at 121 °C for 20 min at 1.5 atm pressure.

The plant material was kept in a growth room under temperature of 25 ± 2 °C, photoperiod of 16 h and luminous intensity of 100 μmol.m^−2^.s^−1^ supplied by LED lamps (Philips brand, model 920008431). The seeds remained in these conditions until complete germination and reached a height of approximately 6 cm, to then be used as sources of explants for the induction of somatic embryogenesis.

### Somatic embryogenesis

#### Somatic embryogenesis establishment

Leaf segments from plants germinated in vitro were used as explants for the induction of callogenesis of *P. hispidinervum*. For experiment 1, explants with about 0.5 cm^2^, coming from different accesses were cultivated in Petri dishes (15 × 90 mm) containing MS medium [25] supplemented with α-naphthalenoacetic acid (NAA) (Sigma, St. Louis, MO, USA) and 6-benzylaminopurine (BAP) (Sigma, St. Louis, MO, USA) in different combinations, plus 30 g/l sucrose and 2.5 g/l Phytagel (Sigma, St. Louis, MO, USA). For experiment 2, explants with about 1 cm^2^, coming from five different *Piper hispidinervum* accesses (PH17, PH21, PH28, PH37, and PH39) were cultivated in Petri dishes (15 × 90 mm) containing MS medium plus 5 mg/l NAA + 2.5 mg/l BAP, 30 g/l sucrose and 2.5 g/l Phytagel. The pH of the media was adjusted to 5.8 ± 0.1 prior to the addition of the gelling agent and the media were sterilized as mentioned in the previous topic.

The leaf segments were inoculated in a laminar flow chamber with the abaxial face in contact with the culture medium and cultivated in the dark in an incubator chamber, type Biochemical Oxygen Demand (B.O.D.) (Percival brand, model I-30NL), with temperature at 25 °C ± 2 °C, for 60 days (experiment 1) and 80 days (experiment 2). After 30 and 60 days of cultivation, the percentage of primary calluses (initial calluses without embryogenic potential) formed for experiment 1 and, after 40 days, for experiment 2, was evaluated. After 80 days of cultivation, the percentage of embryogenic calluses from experiment 2 was evaluated.

The calluses obtained were transferred to MS medium supplemented with 10 mg/l NAA + 2.5 mg/BAP. All media were added 30 g/l sucrose and 2.5 g/l Phytagel. The calluses were kept in the aforementioned environmental conditions and, after 45 days, the percentage of somatic embryos-like structures was evaluated.

### Germination of somatic embryos-like structures and acclimatization of plants

The obtained somatic embryos-like structures were inoculated in Petri dishes with MS medium, plus 30 g/l sucrose. The somatic embryos-like structures were kept in growth room with temperature of 25 °C ± 2 °C and photoperiod of 16 h, for at least 45 days, until the emission of the meristems.

After the germination of somatic embryos-like structures and plant growths, the acclimatization process was carried out [24]. After washing in running water to eliminate the culture medium, the plants were inoculated in plastic cups of 500 mL containing commercial substrate Bioplant®, which were accommodated in trays, covered with plastic bags and taken for incubation for 10 days in incubator chamber (Percival, model I-30NL), with temperature of 25 °C and photoperiod of 16 h. After acclimatization, the surviving plants were transferred to greenhouse to complete growth.

### Experimental design and statistical analysis

An entirely randomized experimental design was adopted for both experiments. The first experiment consisted of five treatments (combinations of NAA and BAP), with five repetitions with five explants each. The second experiment consisted of five treatments (PH17, PH21, PH28, PH37, and PH39 accesses), with six repetitions with five explants each. The data were submitted to the analysis of variance and the means compared by the Scott-Knott Test at the level of 5% significance, using the Sisvar 4.0 program [26].

### Morphological and anatomical characterization of calluses and somatic embryos-like structures

For anatomical characterization, samples of foliar explants with primary calluses, embryogenic calluses and somatic embryos-like structures of *P. hispidinervum* were taken. Embryos were fixed in Karnovsky [27] (paraformaldehyde 2%, glutaraldehyde 2.5%, and cacodylate buffer 0.2 m pH 7.2) for 24 h in the dissector, under vacuum. The material was dehydrated in increasing ethyl series (30–100%) and included in methacrylate (Historesin, Leica), prepared according to the manufacturer’s instruction.

The blocks were cut longitudinally into sections of 5 μm thick in a rotary microtome (Leica®, RM212RT, Buffalo Grove, IL). The obtained sections were stained with toluidine blue in acid pH [28]. The observations and photographic documentation were made on an Olympus microscope (AX70), equipped with Leica camera and software for image capture. Three replicates were used for each type of sample.

### Analysis of regenerated plants by flow cytometry

For this analysis, approximately 30–50 mg of leaves were used from young *P. hispidinervum* plants regenerated by somatic embryogenesis, from *P. hispidinervum* plants cultivated in greenhouse from seed germination (mother plants) and from the external reference standard–pea (*Pisum sativum* L.).

To obtain the nuclei suspension, the plant material was initially crushed in 1.0 ml of Marie extraction buffer [29], already added to the Petri dish in the presence of ice. Then, the crushed tissue in suspension was vacuumed with the aid of Pasteur’s pipette and filtered in 41 μm mesh (Millipore). Finally, the nuclei suspension was stained with 25 μL of a solution of 1 mg/1 mL of propidium iodide.

In each sample, at least 20,000 nuclei were analyzed regarding fluorescence emission, for relative quantification of nuclear DNA. Histograms were obtained on the Accuri C6 cytometer (Becton Dickinson) and analyzed in the Accuri CFlow Plus BD software. The mean nuclear DNA content, in pictograms (pg), was measured by the equation: DNA amount (pg) = (position of the G1 peak of the sample/position of the G1 peak of the reference standard) x reference DNA [30]. Flow cytometry analysis was performed on 30 randomly selected plants, each repeat being repeated three times.

## Results and discussion

### Somatic embryogenesis establishment

**Fig. 1:**
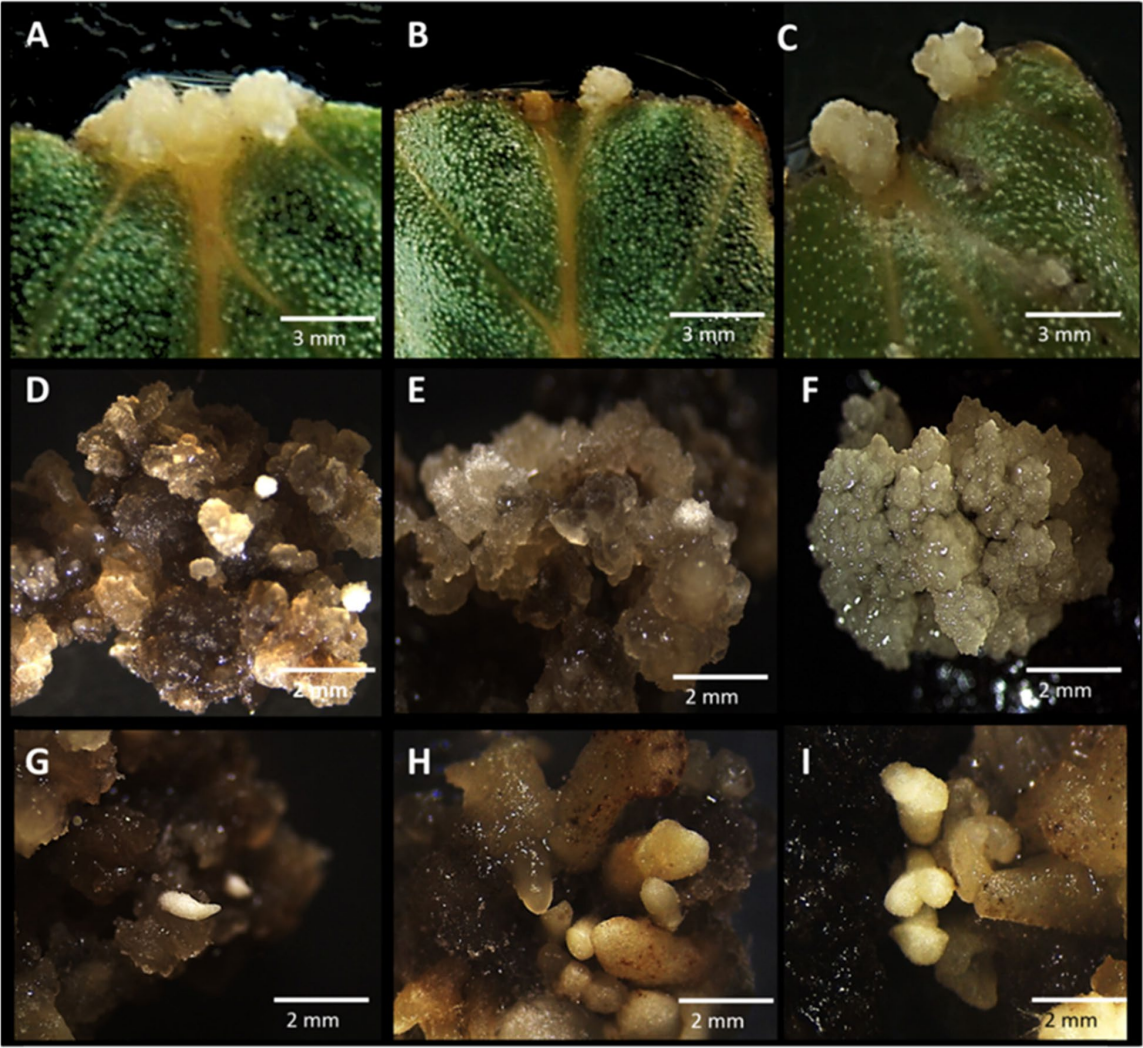
Formation of primary calluses, embryogenic calluses, and somatic embryos-like structures in different accesses of *Piper hispidinervum*. **A**–**C** Emergence of primary calluses, after 40 days of cultivation. **D**–**F** Embryogenic calluses obtained after 80 days cultivation. **G**–**I** Differentiated somatic embryos-like structures after 125 days of cultivation

After 30 days of cultivation, regardless of the treatment tested, the formation of primary calluses with white color and friable consistency was observed, appearing mainly on the leaf ribs and in the regions of section of the explants (Fig. 1). According to statistical analysis, the treatments 2.5 mg/l BAP and 5 mg/l NAA + 2.5 mg/l BAP provided each, after 60 days of cultivation, 32% of primary callus formation (Table 1). Significantly higher percentage of primary calluses, mean value of 87%, was obtained by [24] in leaf segments of *P. aduncum* also cultivated in medium with 5 mg/l NAA + 2.5 mg/l BAP.

**Table 1:** Callus formation from leaf segments of *Piper hispidinervum* under the effect of different combinations of growth regulators, α-naphtalenoacetic acid (NAA), and 6-benzylaminopurine (BAP), aiming at somatic embryogenesis, after 30 and 60 days of cultivation

	Cultivation time	
NAA + BAP (mg L^−1^)	30 days	60 days
0.0 + 0.0	0.0 cA*	0.0 cA
5.0 + 0.0	0.0 cA	0.0 cA
10.0 + 0.0	0.0 cA	0.0 cA
0.0 + 2.5	4.0 bA	32.0 aB
5.0 + 2.5	12.0 aA	32.0 aB
10.0 + 2.5	12.0 aA	12.0 bA
Average	5.0 A	20.6 B

*Means followed by the same letter (uppercase for horizontal comparison and lowercase for vertical comparison) do not differ statistically by the Scott-Knott test at 5% significance

As observed in Table 1, callus formation was not observed from explants cultivated in medium devoid of BAP. This regulator, commonly associated with cell division [31], is, according to the citations of the last 5 years [32], the most widely used in vitro cytokinin, with 31% of citations, possibly due to your effectiveness and accessibility. Singh et al. [33], for example, obtained up to 98.1% of calluses in leaf segments of *Sapindus mukorossi* in medium supplemented with 8.88 μM BAP. The efficiency of BAP in the formation of calluses was also mentioned by Patel et al. [34], in *Curculigo orchioides*, Ren et al. [35] in *Griffinia liboniana*, and Yusuf et al. [36] in *Piper colubrinum*.

Differently of the observed for us, Costa et al. [37] verified the formation of calluses from leaf segments of *P. hipidinervum* in medium supplemented only with NAA, with high percentages observed in the concentrations of 2.5 and 5 mg/l. Valle [38] obtained a higher percentage of friable calluses, also in foliar tissues of this species, in medium supplemented with 2,4-D and BAP. These differences in the requirement for different growth regulators, combined or not, may be related to genotypic variations.

There are several reports of the combination NAA and BAP in the induction of calluses, with results varying according to concentration, species and explant, among other factors. For example, the combination of this auxin and cytokinin provided a high percentage of calluses in foliar explants of *Panax vietnamensis* [39], but it was not efficient for the induction of calluses in *Dioscorea* spp. [40]. This variability in responses should probably be related to the interaction with endogenous hormones in tissues and a possible specificity of the regulator/hormone with receptors and/or co-receptors/effectors, which may vary depending on tissue, subcellular compartment and environmental conditions [41].

**Fig. 2:**
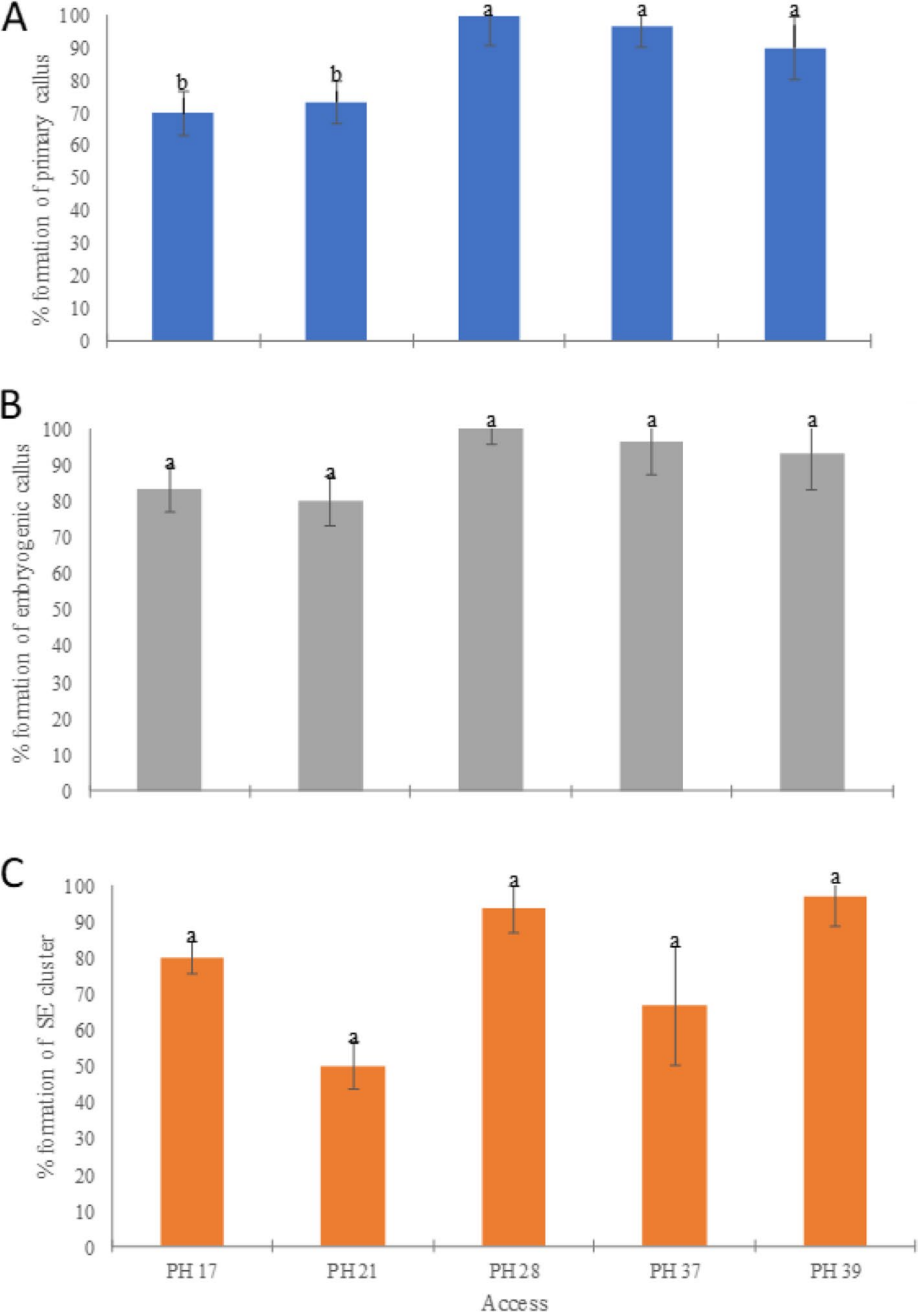
Somatic embryogenesis from different accesses (PH17, PH21, PH28, PH37, and PH39) of *Piper hispidinervum*. **A** Formation of primary calluses. **B** Formation of embryogenic calluses. **C** Formation of somatic embryos-like structures (SE) in embryogenic calluses. The bars represent the standard errors. Means followed by the same letter do not differ statistically from each other by the Scott-Knott test at 5% significance

Once the best treatment for callus induction from foliar explants of *Piper hispidinervum* was defined, the responsiveness of different genotypes to somatic embryogenesis was evaluated. After 40 days of cultivation in medium with 5 mg/l NAA + 2.5 mg/l BAP, the results of the evaluations showed significant differences with regard to the formation of primary calluses (Figs. 1A–C and 2A). Accesses PH28, PH37, and PH39 presented the highest percentages, with 100, 96, and 90% of formation, respectively (Fig. 2A).

Subsequently, the primary calluses were transferred to the somatic embryo formation medium, supplemented with a higher concentration of NAA (10 mg/l), which provided gradual oxidation of the primary calluses concomitantly with the formation of embryogenic masses. The embryogenic calluses had no defined shape and exhibited a texture ranging from friable to watery and beige and/or brown coloration (Fig. 1D-F). Morphologically, the friable calluses obtained here resemble those obtained by [37], also derived from leaf segments of *P. hispidinervum*.

In general, auxins are the growth regulators commonly used in the transition from somatic to embryogenic cells, and may or may not be combined with low concentrations of cytokinins [42]. Among the most used auxins in the induction of somatic embryogenesis, 2,4-D and NAA stand out [43]. In some embryogenic systems, such as in *Medicago truncatula,* the auxin alone (NAA) is not sufficient for the induction of embryogenesis, however, in conjunction with cytokinin (BAP), the process happens [44]. Similarly, the interaction between these two regulators was fundamental for the formation of somatic embryos of *Portulaca oleracea* [45] and *Cucumis melo* [46].

As for the percentage of embryogenic calluses (Fig. 2B) and the percentage of somatic embryos-like structures (Fig. 2C), the five accesses tested did not differ, with mean value of 90.6% and 77.3%, respectively. These results indicate that the combination of NAA and BAP is effective in the induction of somatic embryos-like structures of *P. hispidinervum*, regardless of the access tested, and that foliar explants are efficient for the development of a somatic embryogenesis protocol of the species. In this work, asynchronicity (Fig. 1G-I) was observed in the formation of somatic embryos-like structures, not different from that reported for other species [34, 47, 48], including of the same genera [37, 49].

Despite the existence in the literature of several reports regarding the variability in the embryogenic response of different genotypes of the same species using the same induction conditions [46, 50–53], the same was not observed in this work. Differences were found among the genotypes tested only related to the formation of primary (non-embryogenic) calluses, with the lowest rate obtained still considered relatively high (genotype PH 17: 70%) (Fig. 2A).

According to [43], the beginning of the somatic embryogenesis process is a highly hereditary feature, which opens the possibility that a greater number of efficient genotypes for the induction of this morphogenetic route can be made available through conventional breeding. According to [54], if at least one of the parents with high efficiency in the induction of somatic embryogenesis is included in each breeding pair, there is the possibility of obtaining families with reasonably high initiation rates of somatic embryogenesis. In this context, the relevance of investigating the embryogenic capacity of different higher genotypes of *Piper hispidinervum* is noted.

It is noteworthy, the apparently positive role of oxidation of primary calluses in the induction of somatic embryogenesis of *P. hispidinervum*, previously reported also in *P. aduncum* [24]*.* The formation of somatic embryos from oxidized calluses is not a feature limited to species of the genus *Piper*. In *Coffea canephora* [55], *Hevea brasiliensis* [56, 57], *Arbutus unedo* [58], *Fraxinus mandshurica* [59–61], and *Eucalyptus grandis x E. urophylla* [62], the darkening of explants has been positively related to somatic embryogenesis and, in some cases, considered a prerequisite for the occurrence of this morphogenetic pathway. According to [60], occurrence of darkening of explants of *Fraxinus mandshurica* was not only a consequence of the oxidation of phenolic compounds, but mainly of stress-related processes, which in turn are involved with programmed cell death (PCD) and with the induction of somatic embryogenesis.

Some authors consider somatic embryogenesis as an adaptation response of the plant genome to the stresses inherent to in vitro cultivation [63, 64]. According to [65], when the degree of stress exceeds cellular tolerance, the cells collapse and die, on the other hand, when the level of stress is lower, there is an increase in metabolic activity and induction of the adaptation process, including gene expression reprogramming and cell reorganization. It is emphasized that the establishment of somatic embryogenesis is characterized by the expression of numerous genes related to different stresses, especially those encoding transcription factors involved in hormonal signaling and cell differentiation [66]. Some authors have investigated the positive involvement of reactive oxygen species (ROS) in somatic embryogenesis [57, 67], which are generally produced under stress conditions [68] and associated with irreversible cellular damage [69]). The non-occurrence of oxidized calluses by overaccumulation of glutathione, an anti-ROS enzyme, reduced the efficiency of somatic embryogenesis in *Hevea brasiliensis* [57].

According to [70] and, later, Corredoira et al. [71] associated the presence of darkened exudates in embryogenic cultures of *E. nitens* with a possible role of protection against unfavorable environmental conditions during cultivation. More recently, Silva-Cardoso et al. [72] also raised a similar hypothesis when describing the somatic embryogenesis of *Syagrus oleracea*. In this context, the effects of oxidation on the acquisition of competence for the somatic embryogenesis route in *P. hispidinervum* deserves further investigation to elucidate its role.

### Germination of somatic embryos-like structures and acclimatization of plants

**Fig. 3:**
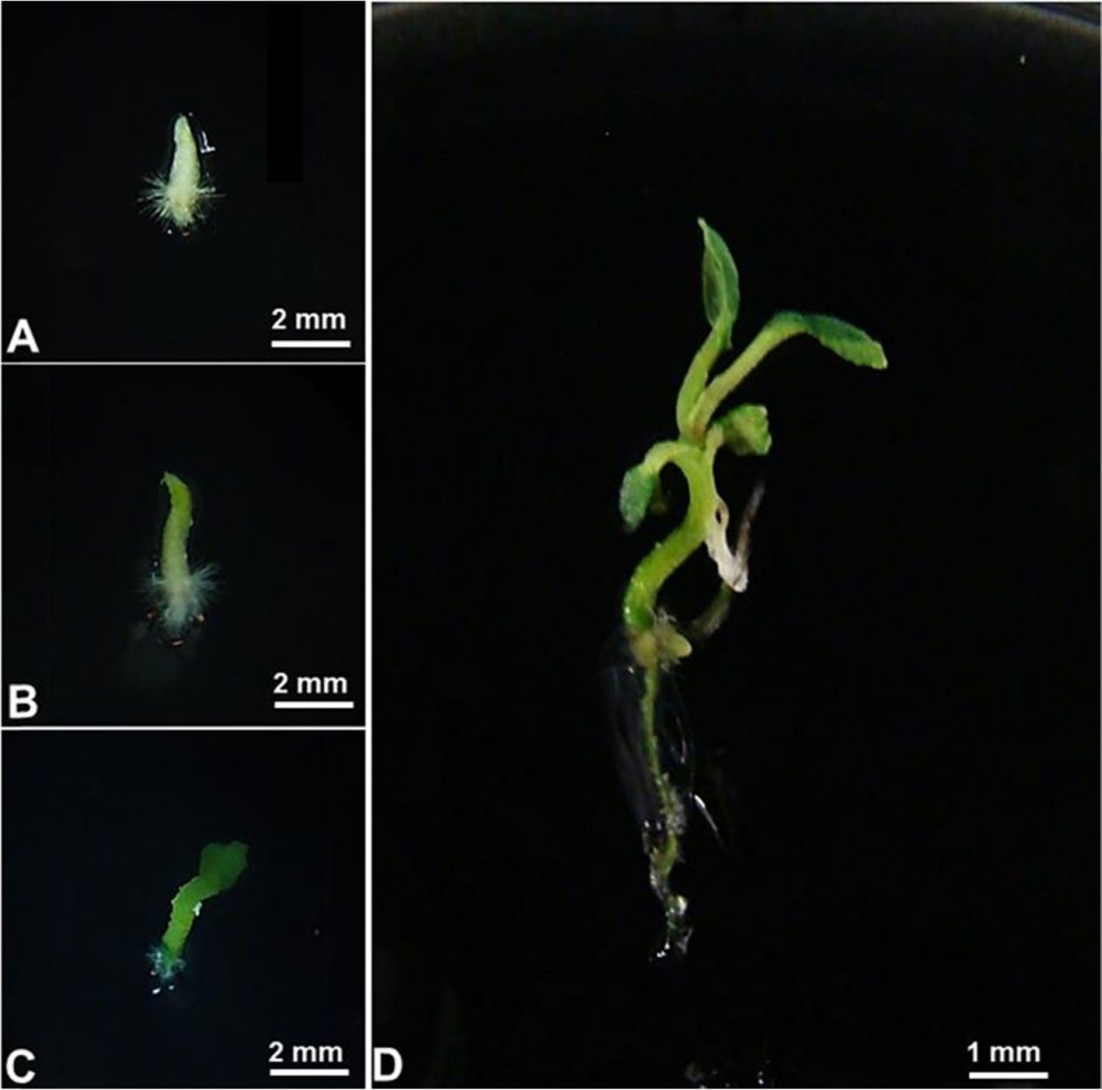
Stages of regeneration of *Piper hispidinervum* somatic embryos-like structures*.* **A** Newly inoculated somatic embryos-like structures. **B**–**D** Development of aerial part and root system. **D** Complete plant

The conversion of somatic embryos into plantlets is an important step in somatic embryogenesis process. After 15 days of inoculation in culture medium with MS salts and vitamins devoid of regulators, the somatic embryos-like structures *P. hispidinervum* began to develop (Fig. 3A). Initially, there was the emergence of an axial root presenting area of piliferous layer and root cap (Fig. 3B), followed by the development of the aerial part (Fig. 3C).

**Fig. 4:**
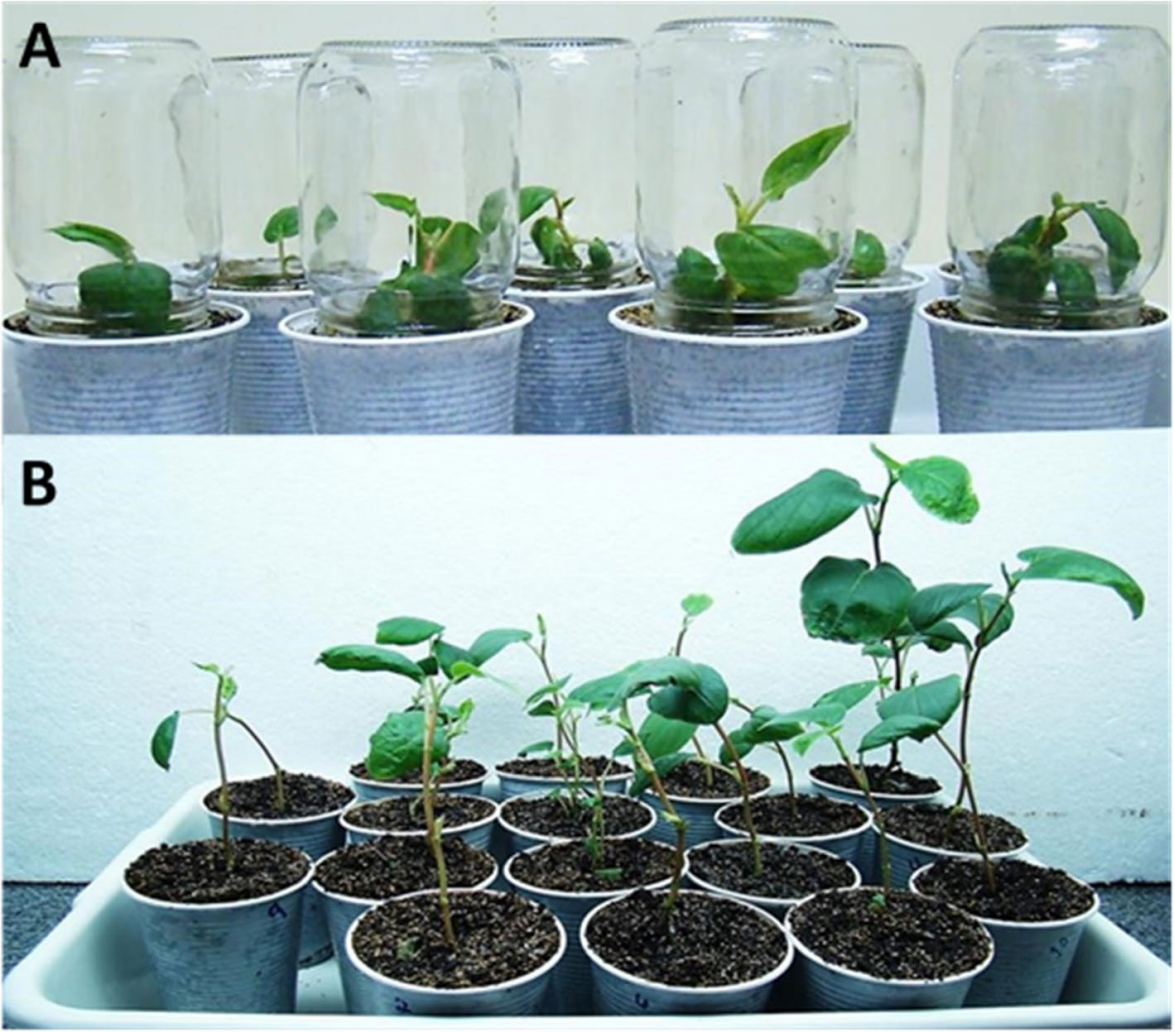
Plants regenerated by somatic embryogenesis of *Piper hispidinervum*. **A** Plants in B.O.D. for acclimatization. **B** Plants in greenhouse post-acclimatization

After 45 days in germination medium, the *P. hispidinervum* plants already presented development of root system, stem and lateral buds ideal for acclimatization phase (Fig. 3D), aiming at adaptation to in vitro post-cultivation humidity and temperature conditions (Fig. 4A). The plants transferred to greenhouse, previously acclimatized in incubator chamber, showed 100% survival (Fig. 4B). These results indicate high efficiency of the somatic embryogenesis process of *P. hispidinervum* induced by NAA and BAP, similar to the one mentioned for *P. aduncum* [24].

### Morphoanatomical characterization

Somatic embryogenesis induction protocols from different explants can be developed or optimized through the use of knowledge of anatomical and histochemical aspects related to the origin of somatic embryos, as well as the sequence of events that lead to their development [43]. In addition, anatomical analysis allow the differentiation between somatic embryogenesis and organogenesis routes, which may, depending on the species and general cultivation conditions, occur in the same system. Given its importance, histological analysis of primary calluses, embryogenic calluses, and somatic embryos-like structures of *P. hispidinervum* were performed.

**Fig. 5:**
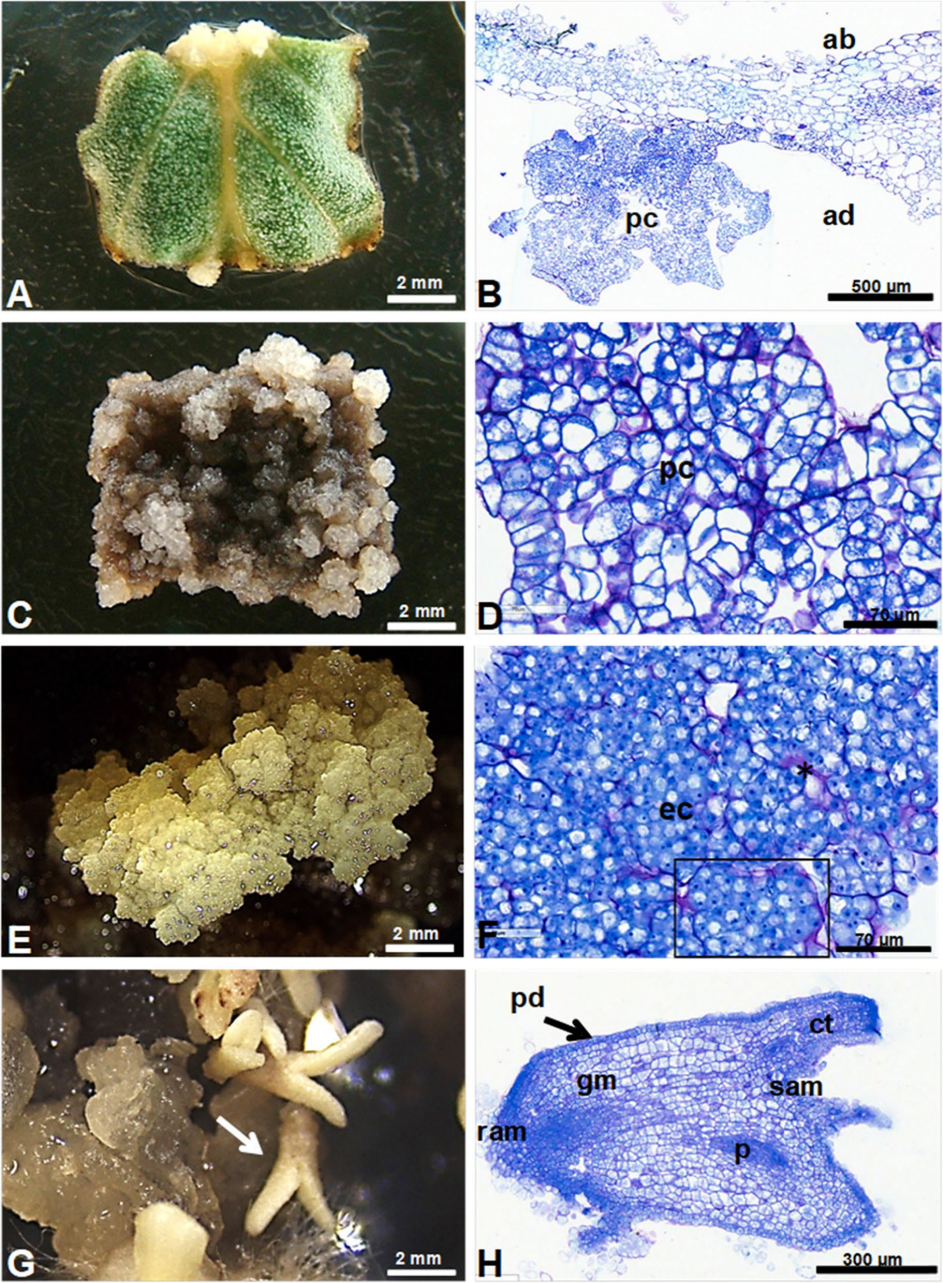
Morphoanatomical characterization of somatic embryogenesis of *Piper hispidinervum* from foliar explants. **A** Foliar explant with primary callus after 40 days in induction medium (MS medium with 2.5 mg L^−1^ BAP + 5 mg L^−1^ NAA). **B** Cross-section of leaf evidencing formation of primary callus. **C** Primary callus. **D** Anatomical section of primary callus. **E** Embryogenic callus. **F** Histology of embryogenic callus; note clustering of cells (rectangle) and purple mucilage (*). **G** Embryogenic callus and somatic embryos-like structures (arrow). **H** Anatomical cut of somatic embryos-like structures; observe cotyledons, apical meristems, procambium, and protoderm (arrow). Abbreviations: (ab) abaxial, (ad) adaxial, (ec) embryogenic callus, (gm) ground meristem, (pc) primary callus, (pd) protoderm, (p) procambium, (ram) root apical meristem and (sam) shoot apical meristem

At 40 days of cultivation, primary calluses were visualized, mainly on the ribs of the adaxial region of the explant (Fig. 5A). The formation of calluses in the adjacencies of vascular tissues has been commonly mentioned in the literature [73–77] and may be related to phloem proximity, which is responsible for the transport of hormones [78] involved with cell division, and composed of responsive vascular stem cells [76, 79].

The primary calluses presented cells with parenchymatous characteristics, with visible nuclei, large vacuoles, thin cytoplasm and visible cell spaces (Fig. 5A-D). Such characteristics resemble those mentioned for primary calluses of *P. aduncum* [24]. Some smaller cells, evident nuclei, and dense cytoplasm (Fig. 5D) were also found at the edge of the primary calluses, probably cells competent for the subsequent formation of embryogenic calluses. Recently, it were reported morphoanatomic characteristics of two types of primary calluses, one with competence for somatic embryogenesis and the other without [77]. The first was characterized by parenchymatous cells with reduced intercellular spaces, while the second type also presented parenchymatous cells, however, with large intercellular spaces.

In the anatomical sections of the embryogenic calluses Fig. 5E, regions with cells with meristematic, isodiametric characteristics, with dense cytoplasm, evident nuclei, thinner cell walls and absence of intercellular spaces were observed (Fig. 5F). Several cells presented two nuclei in the same cytoplasm, showing the cytokinesis not yet completed in the cell division process. These characteristics have been reported in other embryogenic systems [72, 80, 81].

We also observed clustering of these cells, forming aggregates separated by apparently thicker cell walls and surrounded by purple-colored mucilage, typical characteristics of friable calluses (Fig. 5F). According to [82], mucilaginous substances fill the spaces between cell aggregates, which facilitates the diffusion of nutrients and metabolites into friable calluses. This type of callus has often been considered embryogenic in different species [77, 83] and is a prerequisite for the establishment of cell suspension cultures [84].

At 125 days of cultivation, somatic embryos-like structures were observed at different stages of development, including the cotyledonar (Fig. 5G, H), and without any vascular connection with the explant of origin. Somatic embryos-like structures at this stage exhibited protoderm composed of juxtaposed cells, with evident nuclei and tabular format. Below these, some layers of cells with a larger diameter were observed, which characterize a fundamental meristem. In the center, narrow and elongated cells that make up the procambium were observed. Constituting the basal layer of the embryos, the root apex was noted, formed by cells with reduced size compared to the other cells of the embryo, with evident nuclei, dense cytoplasm and isodiametric format. Distinct cotyledons and the apical promeristem were also observed (Fig. 5H). The histological analysis performed did not allow to infer on the unicellular or multicellular origin of the somatic embryos-like structures obtained.

### Analysis of the nuclear DNA content of plants regenerated by flow cytometry

**Fig. 6:**
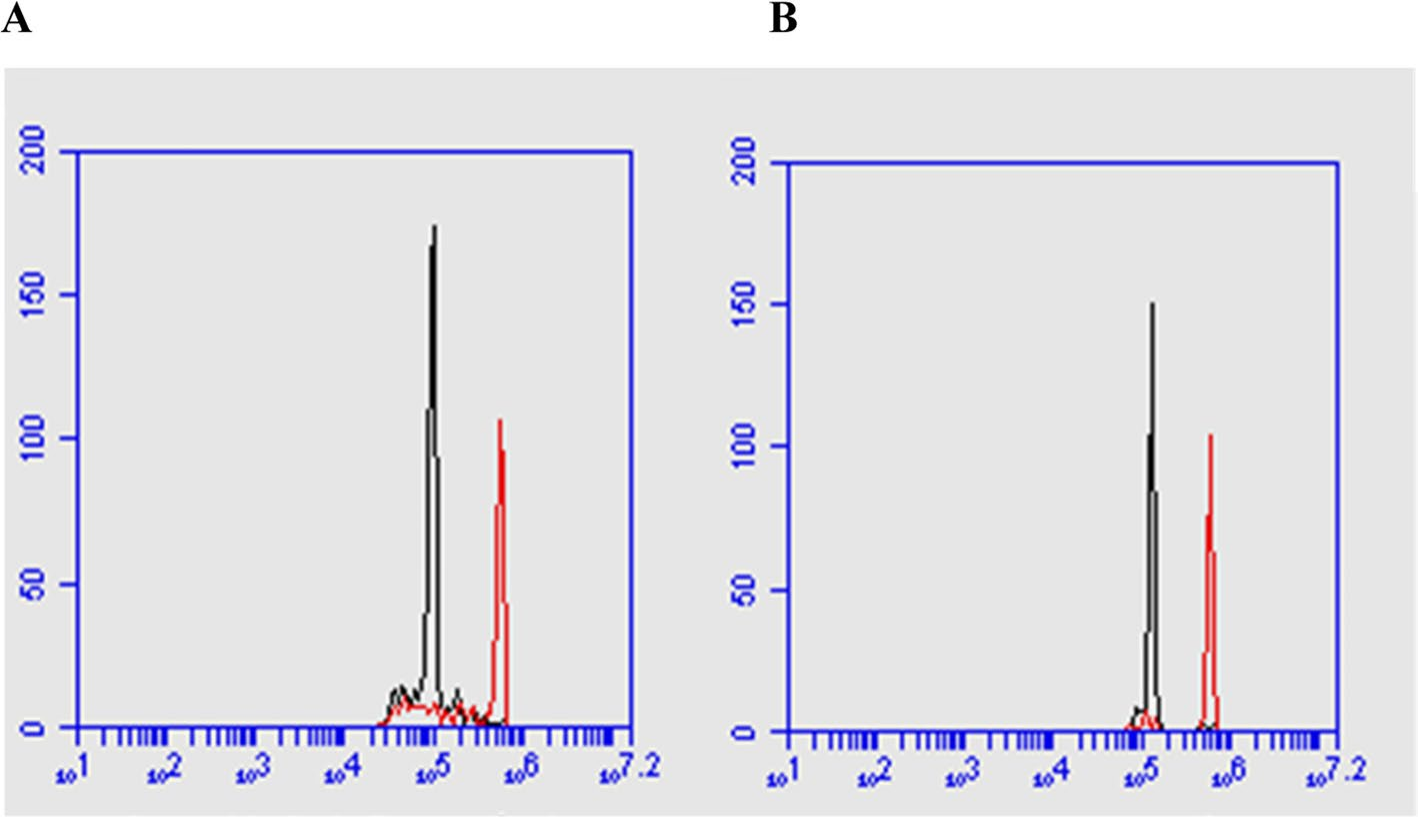
Histograms relating to flow cytometry analysis of the DNA content in *Pepper hispidinervum*. The first peak corresponds to *P. hispidinervum* and the second peak corresponds to the pea reference standard (*Pisum sativum* L.). **A** Plants regenerated via somatic embryogenesis and **B** Plants grown in greenhouse from seed germination

The analysis of young leaves of *P. hispidinervum* resulted in tapered G1 DNA peaks, with good resolution, which indicate reliability of the results presented. Representative histograms of the analysis can be viewed in Fig. 6.

Regarding specifically the nuclear genome size of *P. hispidinervum* plants from somatic embryogenesis and those from greenhouse from seed germination, the estimated content of 1.83 ± 0.02 pg and 1.86 ± 0.05 pg, respectively, was observed on average. The results indicated that the genome size did not differ significantly among plants, thus demonstrating the genomic stability of the plants regenerated via somatic embryogenesis (Table 2).

**Table 2:** Relative DNA content (pg) between in vitro plants derived from somatic embryogenesis and plants grown in greenhouse from *Piper hispidinervum* seeds

Plants	Peak average	Nuclear DNA content	Genome 1C (Mpb)	CV(%)
Somatic embryogenesis	126.19^x^	1.8329 ± 0.02 a^y^	891,88^z^	4.7^v^
Seeds (greenhouse)	123.32	1.8666 ± 0.05 a	912,76	4.6

^x^Calculated from the mean peak G0/G1 of *P. hispidinervum* in relation to the external reference standard *Pisum sativum* L.

^y^Means followed by the same letter, belong to the same group, by the Tukey test at the level of 5% probability

^z^Mean genome size 1C, where 1 pg DNA = 978 Mpb (Dolezel et al*.* 2003)

^v^Mean of the coefficient of variation of peak G0/G1

The quantification of DNA for species of *Piper* had already been performed by Samuel et al. [85]. The authors analyzed five species of *Piper* from the old world (two varieties of *P. nigrum*: 4×, 8×; *P. longum*: 4×; *P. betle*: 4×; *P. silvestre*: 4×; *P. argyrophyllum*: 4×) and four of the new world (*P. apiculatum*: 2×; *P. cernum*: 2×; *P. arboreum*: 2×; *P. ornatum*: 4×) including among them cultivated and wild species. The quantification was performed by Feulgen densitometry and the nuclei analysis was in G2 (4C). According to the authors, wild species tend to have higher DNA content than cultivated species. More recently, Phurailatpam et al. [86], using the flow cytometry technique, identified differences in ploidy between plants of different sexes of *P. betle* (females: 4× and males: 3×).

Flow cytometry has been used to evaluate the genetic stability of regenerants obtained by different in vitro cultivation techniques, including by somatic embryogenesis. Factors such as genotype, explant source, growth regulators, age of cultivation [87], and stress inherent to in vitro cultivation during somatic embryogenesis itself [21] may affect the genetic stability of regenerated plants. However, in this study, there was no variation in the nuclear DNA content of nuclear plants. *P. hispidinervum* from the germination of somatic embryos-like structures.

Stability in genome size has also been reported in plants regenerated by somatic embryogenesis of *Coriandrum sativum* [88], *Hibiscus sabdariffa* [89], *Milkweed* spp. [90], *Juglans directed* [91], and *Rauvolfia serpentina* [92] evaluated by flow cytometry.

On the other hand, Raji et al. [46] found that 7% of *Cucumis melo* plants regenerated via somatic embryogenesis were tetraploid with respect to the mother plant, considered diploid. Despite the identification of this somaclonal variation, the authors consider that the occurrence of similarity with respect to nuclear DNA content in 93% of regenerated plants is an indication of high genetic stability. There are other reports of significant differences in the DNA content between plants cultivated ex vitro and somatic embryogenesis, as reported by Acanda et al. [93] who obtained coefficient of variation of 5.6% in the content of vine DNA (*Vitis vinifera* L. cv. Mencía) obtained from somatic embryogenesis.

In these studies of genomic stability of in vitro cultivations through the flow cytometry technique, the coefficients of variation are essential indications of data reliability and the estimation of the mean DNA content. Marie and Brown [31] proposed coefficients of variation from 1 to 2% for high-quality results, and around 3% for routine results. Galbraith et al. [94] defined the value of 5% for the coefficients of variation, as a criterion for acceptance of DNA content estimates.

The coefficients of variation observed in this work were less than 5%, and in general, ensure the stability and reliability of the somatic embryogenesis method in the production of *P. hispidinervum* clones.

## Conclusions

In this study, we reported an efficient and unprecedented protocol to regenerate plants via somatic embryogenesis, from foliar explants of superior genotypes of *P. hispidinervum*, in medium supplemented with NAA and BAP. Flow cytometry analysis did not identify variation between plants derived from somatic embryogenesis in relation to plants from seed germination, which supports the use of this protocol for clonal production, germplasm conservation, and genetic transformation of the species.

## Data Availability

The datasets generated during and/or analyzed during the current study are available from the corresponding author on reasonable request.
